# Supplementary material for: Research on defect detection of bottle cap interior based on low-angle and large divergence angle vision system
Source: PLoS One. 2024 May 31;19(5):e0303744. doi: 10.1371/journal.pone.0303744 (PMC11142609; doi:10.1371/journal.pone.0303744)
Supplement: Attachment — Submitted filename: Response to Reviewers.docx [file pone.0303744.s002.docx]

**Original Manuscript ID:** PONE-D-24-01223

**Original Article Title: “**Research on defect detection of bottle cap interior based on low-angle and large divergence angle vision system**”**

**To:** PLOS ONE Editor

**Re:** Response to reviewers

Dear Editor,

Thank you for allowing a resubmission of our manuscript, with an opportunity to address the reviewers’ comments.

We are uploading (a) our point-by-point response to the comments (“Response to Reviewers”), (b) an updated manuscript with yellow highlighting indicating changes (“Revised Manuscript with Track Changes*”*), and (c) a clean updated manuscript without highlights (“Manuscript”)*.*

Best regards,

Chen Li et al.

**Reviewer#1, Concern # 1: It is important to address whether the luminous intensity of the light source has any impact on the test results, and if so, provide a thorough explanation in the manuscript.**

**Author response:** Thank you for your comments. Comparative experiments with different light source intensities have been added to confirm that the LALDA system works equally well over a wide range of light intensities.

**Author action:** We have supplemented the relevant experiments and updated the manuscript.

**Reviewer#1, Concern # 2: The authors may add more state-of-art computer vision & robot articles in engineering application for the integrity of the manuscript (3D vision technologies for a self-developed structural external crack damage recognition robot; Automation in Construction. Enhanced Precision in Dam Crack Width Measurement: Leveraging Advanced Lightweight Network Identification for Pixel-Level Accuracy; International Journal of Intelligent Systems.).**

**Author response:** Thank you for your comments. We added relevant literature studies.

**Author action:** We have updated the manuscript.

**Reviewer#1, Concern # 3: In the experimental section, the average detection correct rate is reported as 97.3% and compared with the average accuracy results of other deep learning networks in Table 1. However, it's crucial to acknowledge that the average accuracy of the deep learning network is just one type of network evaluation index and may not be directly comparable to the average detection correct rate. This distinction should be carefully considered and appropriately addressed.**

**Author response:** Thank you for your comments. As you mentioned, our study does not adequately take into account the comparison of other evaluation metrics in deep learning, nor does it clearly define the average accuracy. In view of this, we have supplemented the study as follows, and described accordingly in the manuscript: pixel accuracy (PA) and average accuracy (MA) are mainly used to evaluate classification accuracy, but in actual cap production, more attention is paid to the accuracy of defect detection, that is, whether defects can be accurately identified to meet the manufacturer's 95% accuracy requirement (at least 95 out of 100 defective caps can be correctly identified). Therefore, we chose IoU (Intersection over Union) as the evaluation index in the paper to measure accuracy more accurately.

**Author action:** We have updated the manuscript.

**Reviewer, Concern # 4: The paper's detection method and algorithms represent a departure from traditional detection schemes. Therefore, it is advisable to expand the comparison with traditional detection schemes to demonstrate the improvement in the correct detection rate.**

**Author response:** Thank you for your comments. We supplemented the comparison experiments of high-angle light sources and coaxial light sources in the traditional method, and analyzed the existing problems.

**Author action:** We have supplemented the relevant experiments and updated the manuscript.

**Reviewer, Concern # 5:** **Provide a clear definition of "defective caps" to ensure readers have a complete understanding of the terminology used in the study.**

**Author response:** Thank you for your comments. We have made appropriate changes and explanations to the relevant descriptions.

**Author action:** We have updated the manuscript.
